# *Escherichia coli* O157:H7 Super-Shedder and Non-Shedder Feedlot Steers Harbour Distinct Fecal Bacterial Communities

**DOI:** 10.1371/journal.pone.0098115

**Published:** 2014-05-23

**Authors:** Yong Xu, Eric Dugat-Bony, Rahat Zaheer, Lorna Selinger, Ruth Barbieri, Krysty Munns, Tim A. McAllister, L. Brent Selinger

**Affiliations:** 1 Department of Biological Sciences, University of Lethbridge, Lethbridge, Alberta, Canada; 2 AgroParisTech National Institute for Agricultural Research, Thiverval, Grignon France; 3 Agriculture and Agri-Food Canada, Lethbridge Research Centre, Lethbridge, Alberta, Canada; University of Queensland, Australia

## Abstract

*Escherichia coli* O157:H7 is a major foodborne human pathogen causing disease worldwide. Cattle are a major reservoir for this pathogen and those that shed *E. coli* O157:H7 at >10^4^ CFU/g feces have been termed “super-shedders”. A rich microbial community inhabits the mammalian intestinal tract, but it is not known if the structure of this community differs between super-shedder cattle and their non-shedding pen mates. We hypothesized that the super-shedder state is a result of an intestinal dysbiosis of the microbial community and that a “normal” microbiota prevents *E. coli* O157:H7 from reaching super-shedding levels. To address this question, we applied 454 pyrosequencing of bacterial 16S rRNA genes to characterize fecal bacterial communities from 11 super-shedders and 11 contemporary pen mates negative for *E. coli* O157:H7. The dataset was analyzed by using five independent clustering methods to minimize potential biases and to increase confidence in the results. Our analyses collectively indicated significant variations in microbiome composition between super-shedding and non-shedding cattle. Super-shedders exhibited higher bacterial richness and diversity than non-shedders. Furthermore, seventy-two operational taxonomic units, mostly belonging to *Firmicutes* and *Bacteroidetes* phyla, were identified showing differential abundance between these two groups of cattle. The operational taxonomic unit affiliation provides new insight into bacterial populations that are present in feces arising from super-shedders of *E. coli* O157:H7.

## Introduction

*Escherichia coli* O157:H7 is a serotype of enterohemorrhagic *E. coli* (EHEC) that produces shiga-like toxins and is a major public health concern worldwide. Conditions caused by *E. coli* O157:H7 range from mild diarrhea to hemorrhagic colitis and life-threatening hemolytic uremic syndrome [1]. This pathogen typically affects children, the elderly and immunocompromised patients [2]. Cattle are a principal reservoir of *E. coli* O157:H7, which preferentially colonizes the lower gastrointestinal tract (GIT) of cattle and in particular the rectal-anal junction [3], [4]. Cattle can shed *E. coli* O157:H7 heterogeneously, ranging from some individuals that do not shed *E. coli* O157:H7 (non-shedders) to those that shed very high concentrations of the pathogen. Cattle shedding *E. coli* O157:H7 >10^4^ CFU/g of feces have been designated as “super-shedders” [5]. Super-shedders are hypothesized to be responsible for majority of transmission of this bacterium among individuals and its dissemination into the environment. Consequently, they have been proposed as prime targets for mitigation strategies aimed at reducing the incidence and spread of *E. coli* O157:H7 [6].

Currently, very little is known about the factors that contribute to the shedding of *E. coli* O157:H7. Microbial-specific, host-specific and environmental factors likely all play a role in the development of the super-shedding phenomenon. Based on many recent studies on the impact of intestinal microbiota on host well-being, it could be hypothesized that the incidence and shedding of *E. coli* O157:H7 is likely affected not only by characteristics of the strain but also by the nature of the microbiome (i.e., bacteria, fungi and protozoa) within the host's GIT. The GIT microbial community is critical to host health and well-being and it is projected that even minor changes in these populations may cause dramatic shifts that affect livestock productivity [7]. The normal microbiota not only produces necessary nutrients (i.e., vitamins and short chain volatile fatty acids) [8], [9] but also has been implicated in the development of a healthy immune system and the exclusion of enteric pathogens [10]. Recent studies using fecal samples from cattle have revealed a high degree of animal-to-animal variation in the GIT bacterial community [11], [12].

In this study, we hypothesized that the super-shedder condition is a result of intestinal dysbiosis and that a healthy normal microbiota prevents *E. coli* O157:H7 from colonizing the intestinal tract in a manner that leads to the super-shedding state. To test this, we employed Bacterial Tag-Encoded FLX Amplicon Pyrosequencing Analysis (bTEFAP) [13] to generate over 200,000 partial 16S rRNA gene sequences from fecal samples collected from 11 super-shedders (SS cattle) and 11 cotemporary pen mates which were negative for *E. coli* O157:H7 (NS cattle). Sequence data was processed to 1) depict the bacterial community structure for fecal samples from SS cattle, 2) compare the bacterial community profiles for SS and NS cattle and 3) identify the operational taxonomical units (OTUs) differentially present in each group.

## Materials and Methods

### Sample collection and *E. coli* O157:H7 enumeration

All cattle used in this experiment were handled in accordance with the Canadian Council of Animal Care [14] and the protocol (#1120) was reviewed and approved by the nationally accredited Lethbridge Research Centre Animal Care Committee. Crossbred yearling feedlot steers (n = 400), from a single commercial feedlot in southern Alberta were sampled in July of 2011. All cattle were fed a barley-grain based finishing diet. Fecal samples (∼50 g) were collected by rectal palpation, immediately placed on ice, transported to the laboratory and analyzed within 4 h after collection. *E. coli* O157:H7 was enumerated from feces by serially diluting 1 g into 9 mL of phosphate buffered saline and plating 100 µL (in duplicate) dilutions ranging from 10^−1^ to 10^−4^ onto sorbitol MacConkey agar with 2.5 mg/L potassium tellurite and 0.05 mg/L cefixime (CT-SMAC; Dalynn Biologicals, Calgary, AB). Plates were incubated at 37°C for 18 to 24 h and colonies were enumerated using a colony counter (Reichert, Depew, NY). Steers identified with >10^4^ cfu of *E. coli* O157:H7/g of feces were identified as super-shedders (SS). Three representative non-sorbitol fermenting colonies from each sampling point were confirmed to be *E. coli* O157 using the *E. coli* O157 latex test kit (Oxoid Ltd., Basingstoke, Hampshire, UK). Positive agglutination isolates were confirmed by multiplex PCR, whereby template DNA was prepared from a single colony suspended in sterile water and heat lysed. From this, 1 µl of the supernatant was used in PCR with conditions described by Gannon *et al*. [15] to test for the presence of genes specific to the O157:H7 serotype (*vt*, *eaeA*, *fliC*).

When *E. coli* was not detectable by plating, duplicate 1 g subsamples of feces were enriched in 9 mL of modified TSB containing novobiocin (20 mg/L; Sigma-Aldrich Canada Co., Oakville, ON, Canada), bile salts (1.5 g/L; BD – Canada, Mississauga, ON, Canada), dipotassium phosphate (1.5 g/L; Sigma-Aldrich Canada Co.) and TSB (30 g/L; BD – Canada) and incubated for 6 h at 37°C. Enriched samples were then subject to immunomagnetic separation using anti- *E. coli* O157:H7 Dynabeads (Invitrogen, Carlsbad, CA) as per manufacturer's instructions. A 50 µL aliquot of bead-bacteria complex was plated onto CT-SMAC (Dalynn Biologicals) and incubated at 37°C for 18 to 24 h. Three non-sorbitol fermenting clear colonies were randomly selected for latex confirmation and PCR as described above. Cattle that were negative both by enumeration and IMS were classified as non-shedders (NS).

### Bacterial Tag-Encoded FLX Amplicon Pyrosequencing Analysis

Community DNA was extracted from 0.25 g of feces from SS cattle (n = 11) and NS cattle (n = 11) using the QIAamp DNA Stool kit (Qiagen, Toronto, ON, Canada) and analyzed at the Research and Testing Laboratory (Lubbock, Texas, USA) by using bacterial tag-encoded FLX amplicon pyrosequencing (bTEFAP)[13]. Primers that span the hypervariable regions V1 and V3 of the 16S rRNA gene (28F 5′GAGTTTGATCNTGGCTCAG and 519R 5′ GTNTTACNGCGGCKGCTG), were used in sequencing reactions. Sequence coverage was approximately 10,000 sequences per sample.

### Sequence data processing

Raw sequence read data were quality-filtered using Mothur v1.23 [16] to remove sequences less than 200 nucleotides (nt), sequences containing homopolymers >8 nt, mismatches in the barcode or primer, >1 ambiguous nt, or regions in which an average quality score below 30 was obtained using a moving window of 50 nt. The remaining sequences were aligned to the SILVA-based bacterial reference alignment [17] using the Needleman-Wunsch algorithm [18]. Potential chimeric sequences were removed using UCHIME in reference mode [19]. Finally, sequencing noise was reduced by a pre-clustering step implemented in Mothurs v1.23 [16]. Sequence data have been submitted to the short-read archive (Bio Project SRP040473 – accession numbers for individual animal samples sequence data are SRX498462, SRX498463, SRX498466, and SRX498471 – SRX498489).

### Microbial community analysis

Previous studies have shown that alpha and beta diversity analyses are very sensitive to the clustering methods used for binning sequences into OTUs [20], [21]. Therefore, five cluster-based algorithms were used in order to reduce bias: Mothur [16], Esprit-tree [22], Otupipe (http://drive5.com/otupipe/), CROP [23] and UPARSE [24]. These algorithms represent three distinct clustering methods: hierarchical clustering (Mothur, Esprit-tree), greedy heuristic clustering (Otupipe, UPARSE) and Bayesian clustering (CROP). For Mothur an average neighbor algorithm cutoff was used. Default parameters were applied for CROP [23] and Esprit-tree [22]. Two MinSizes were set for Otupipe: MinSize = 2 and MinSize = 4. In order to use the UPARSE pipeline, the qual and fasta files for each sample were combined into fastq files [24]. Sequences were filtered to a fixed length of 180 nt and those with an expected error probability greater than 0.5 were discarded. Chimeras were checked against the “Gold” database based on UCHIME [19], and reads were clustered at 97% sequence identity into operational taxonomic units (OTUs).

For all clustering methods a dissimilarity level of 0.03 was used to bin OTUs, a resolution that corresponds to the species level. To assess similarity among the different methods, two reference OTUs datasets were built by mapping quality-filtered, processed sequences against the Greengenes reference OTUs database [25] downloaded from http://greengenes.lbl.gov/Download/Sequence_Data/Fasta_data_files/Caporaso_Reference_OTUs/gg_otus_4feb2011.tgz using USEARCH [26] with a global identity threshold of 97% and 90%, respectively. Taxonomic assignments of OTUs were determined using the Ribosomal Database Project Bayesian classifier algorithm (version 2.2) [27], with an 80% support cutoff. The most recent taxonomy annotation [28] was used to retrain the ribosomal database project (RDP) classifier.

### Statistical analysis

The pyrotag libraries for each sample were normalized based on subsampling to the lowest number of reads (16; Table 1). The different OTU matrices obtained by the above methods were imported into the R version 2.14 statistical computing environment (http://www.r-project.org/). Subsequent analyses including Good's coverage, Richness and Shannon Diversity Index and visualizations were performed in R using functions from the picante version 1.3 [29], vegan version 2.03 (http://r-forge.r-project.org/projects/vegan/), phyloseq version 1.14 [30] and ggplot2 version 0.8.9 [31]. Bacterial community dissimilarity was quantified using Bray-Curtis distance. The Bray-Curtis distance matrix was used to test if the bacterial communities of non-shedders and super-shedders were statistically different by running the ANOSIM function in the vegan package with 1000 permutations. A non-parametric Wilcoxon rank sum test was implemented by R script with *p*<0.05 and False Discovery Rate (FDR) <25% to identify OTUs with significant differences between non-shedders and super-shedders. The compositional similarity of all samples was visualized using a nonmetric multidimensional scaling (NMDS) ordination.

**Table 1:** Comparison of OTU-binning methods.

Method	No. of OTUs	Pyrotags binned into OTUs	No. of pyrotags for normalization^#^	No. of OTUs after normalization	Correlation value to RefMap97
Mothur	8,664	100%	2,942	6,823	0.77
Esprit-tree	5,701	100%	2,942	4,733	0.79
CROP	1,427	100%	2,942	1,246	0.83
UPARSE	1,112	34.5%	859	941	0.92
Otupipe2*	3,801	83.1%	2,502	3,540	0.91
Otupipe4*	1,111	37.9%	1,091	926	0.96
RefMap90^+^	1,780	84.7%	2,419	1,545	0.94
RefMap97^+^	862	37.8%	968	728	1.00

*Otupipe2: MINSIZE = 2 (i.e., the minimum depth is 2); Otupipe4: MINSIZE = 4. ^+^RefMap90: the sequences were mapped into the reference database with an identity threshold of 90%. The identity threshold was 97% for RefMap97. ^#^ No. of pyrotags with the smallest library for the normalization.

## Results

### *E. coli* O157:H7 shedding levels by feedlot cattle

From sampling 400 cattle at the commercial feedlot, a total of 46 (11.5%) were identified as shedding *E. coli* O157:H7 of which 11 (2.8%) of these cattle were super-shedders (>10^4^ *E. coli* O157:H7 cfu/g feces). *E. coli* O157:H7 counts for the super-shedders ranged from 1.5×10^4^ to 6.5×10^7^ cfu/g feces. Eleven non-shedding pen mates were also selected as the control group.

### General sequencing information and taxonomic affiliation

**Figure 1:**
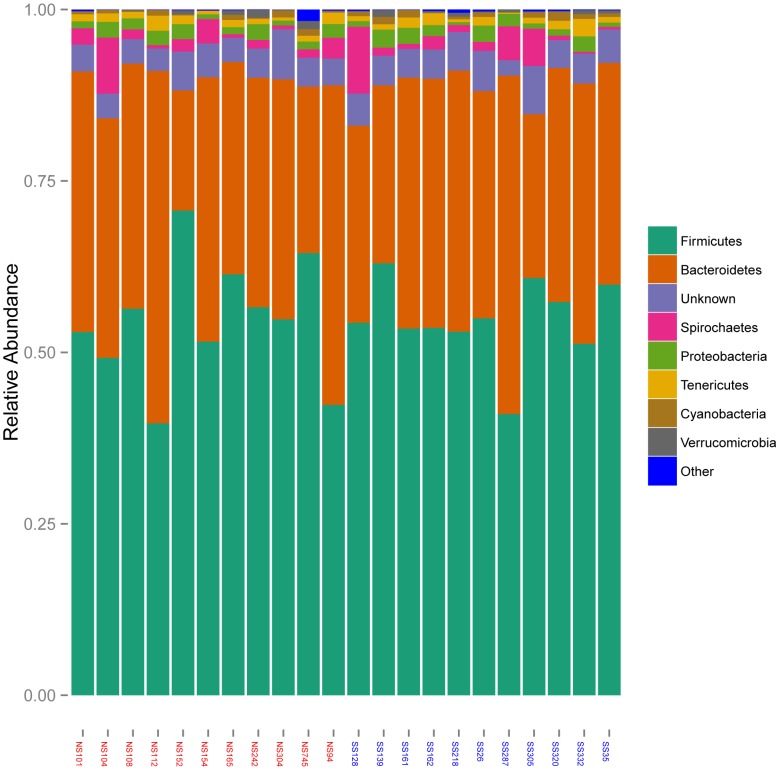
Distribution of bacterial phyla in fecal samples from cattle. The distribution of phyla is based on the relative abundance of 16S rRNA pyrotags from 22 fecal samples from cattle. The taxonomic affiliations were predicted using the RDP classifier with a bootstrap cutoff of 80. Pyrotags that could not be assigned at the phylum rank were classified as “Unknown”. Sample names listed on the x-axis were from *E. coli* O157:H7 negative steers (NS - red) or super-shedders (SS - blue).

Next generation pyrosequencing was used to sequence 16S rRNA genes in metagenomic DNA extracted from fecal samples collected from the 11 SS cattle and 11 NS cattle. In total 236,139 pyrotags were obtained from the 22 fecal metagenomic DNA samples. After removal of low quality reads and chimeras, 112,926 processed sequences remained, with an average of 5,126 reads per sample (ranging from 2,942–7,622) and a mean length of 278 nt (Figure S1). Based on an 80% RDP confidence level, 18 phyla, 33 classes, 57 orders, 77 families and 97 genera were inferred. As expected, the proportion of unclassified sequences increased as the taxonomic grouping decreased. At the phylum level, only 4.4% (4,914) of the sequences remained unclassified, while at the genus level 76.7% (86,591) of the sequences could not be classified. For all samples, the bacterial communities were dominated by *Firmicutes*, accounting for 53.9% of the dataset (60,845/112,926 sequences), followed by *Bacteroidetes* (35.6%; 40,185 sequences), *Spirochaetes* (2.2%; 2,521 sequences) and *Proteobacteria* (1.7%; 1,892 sequences) (Figure 1). The remaining 14 phyla accounted for less than 2.3% of the dataset. At the genus level, *Prevotella* (*Bacteroidetes* phylum) was identified most frequently in fecal samples, accounting for up to 10.5% of the entire dataset. All of the taxa within each of the five taxonomic levels are listed in Table S1. The classifier-based analysis revealed that the composition of bacterial communities differed among steers. Only 5/18 phyla (*Bacteroidetes, Firmicutes, Proteobacteria, Spirochaetes, Tenericutes*), 8/33 classes (*Bacilli, Bacteroidia, Betaproteobacteria, Clostridia, Erysipelotrichi, Gammaproteobacteria, Mollicutes, Spirochaetes*), 5/57 orders (*Bacteroidales, Burkholderiales, Clostridiales, Erysipelotrichales, Spirochaetales*), 12/77 families (*Alcaligenaceae, Bacteroidaceae, Clostridiaceae, Clostridiales FamilyXIII incertae sedis, Erysipelotrichaceae, Lachnospiraceae, Peptococcaceae, Prevotellaceae, Rikenellaceae, Ruminococcaceae, Spirochaetaceae, Veillonellaceae*) and 9/97 genera (*Alistipes, Bacteroides, Clostridium, Eubacterium, Oscillospira, Prevotella, Ruminococcus, Sutterella, Treponema*) were shared by all 22 samples.

### Super-shedder cattle exhibit more diverse fecal bacterial communities than non-shedder cattle

Previous studies have shown that alpha and beta diversities are very sensitive to the clustering methods used for binning sequences into OTUs [20],[21]. Therefore, in this study, five cluster-based algorithms were used to reduce bias. In addition we built two OTU matrices, RefMap97 and RefMap90 by mapping the pyrotags to the Greengenes reference OTUs with global sequence similarities of 97% and 90%, respectively (Table 1). The reference OTU matrices, RefMap97 and RefMap90, are based on the full-length 16S rRNA genes with strict quality control.

Only 38.6% (97% similarity) and 84.3% (90% similarity) of the OTUs could be mapped onto the Greengenes reference OTUs. Five clustering methods that were used to bin pyrotags into OTUs are summarized in Table 1. Three methods including Mothur, Esprit-tree and CROP were able to use all of the processed sequences. In comparison, UPARSE used 34.5% of the processed sequences and Otupipe used 37.6% and 83.1% of the sequences at MinSize parameters of 2 and 4, respectively. The Otupipe MinSize parameter sets the minimum size of a cluster in the precluster process resulting in some sequences being discarded as noise. The total number of OTUs utilized among clustering methods differed by >10 fold.

**Figure 2:**
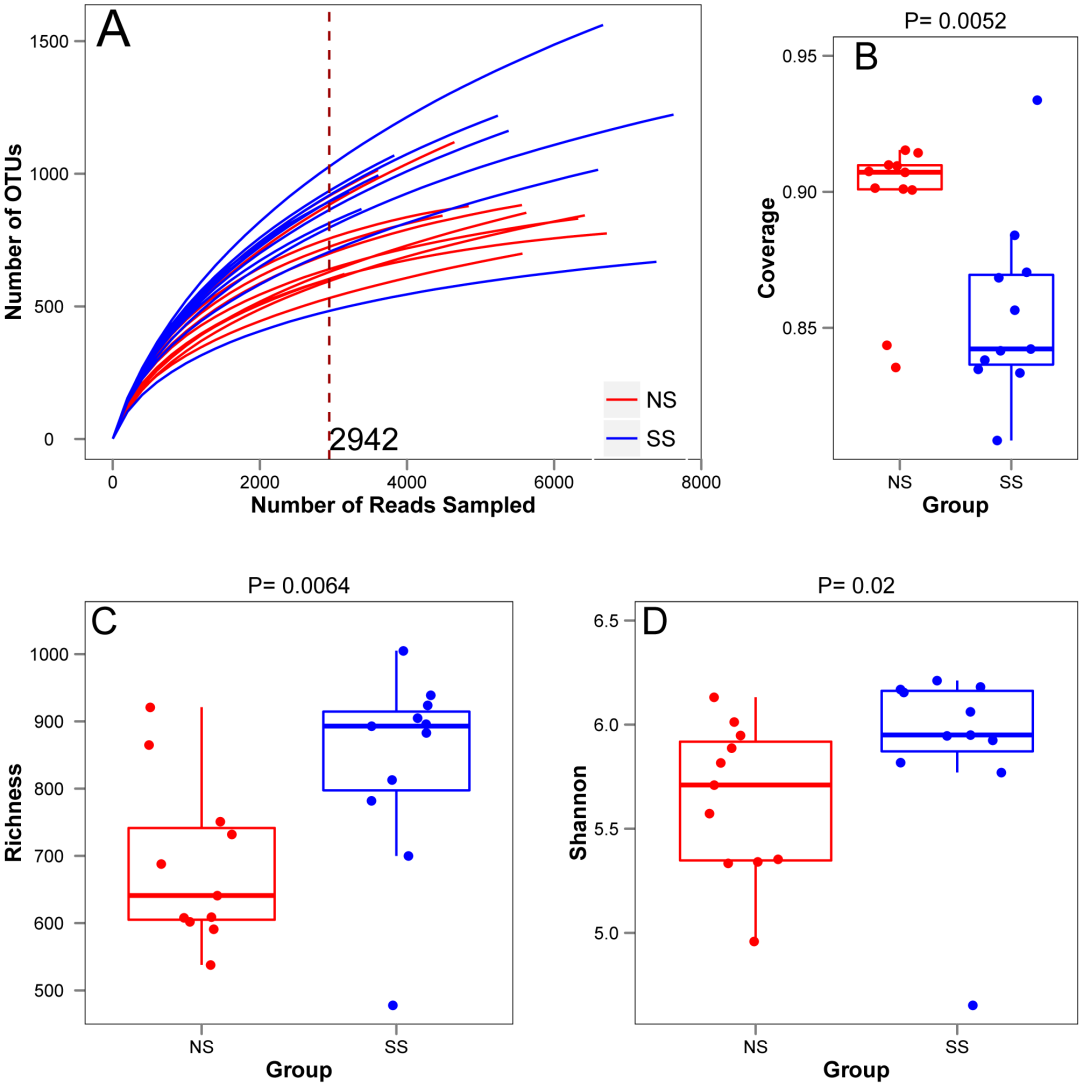
Alpha diversity in cattle fecal samples from super-shedder (SS) and non-shedder steers (NS) as represented by rarefaction curves (A), and boxplots for Good's coverage (B), Richness (C) and Shannon Diversity Index (D) using the Esprit-tree OTU matrix. The number of OTUs was used as a measure of the richness in Fig C. In Fig B, C and D, each box plot summarizes the distribution of the data set. Lower, mid, and upper horizontal lines denote the first quartile, median and the third quartile, respectively. Vertical lines reach the 1.5 interquartile range from the respective quartiles or reach the extreme value, whichever is closer. The remaining clustering methods gave similar results.

The alpha diversity results for each clustering method exhibited similar trends; consequently, only those obtained with the Esprit-tree method are presented in Figure 2. While the rarefaction curves did not plateau (Figure 2A), Good's coverage (which estimates the probability that the next read will belong to an existing OTU) averaged 86.9%±3.3% (mean ± SD) (Figure 2B), indicating that the majority of bacterial diversity was captured with this sequencing effort. The richness (Figure 2C) and Shannon diversity index (Figure 2D) were greater (*p*<0.05, Wilcoxon test) for SS cattle than for NS cattle, regardless of the clustering method used to process the data. Together, these results highlight the fact that fecal bacterial communities of super-shedding cattle are more diverse than those of non-shedders.

### Super-shedders and non-shedders have distinct bacterial community structures

**Figure 3:**
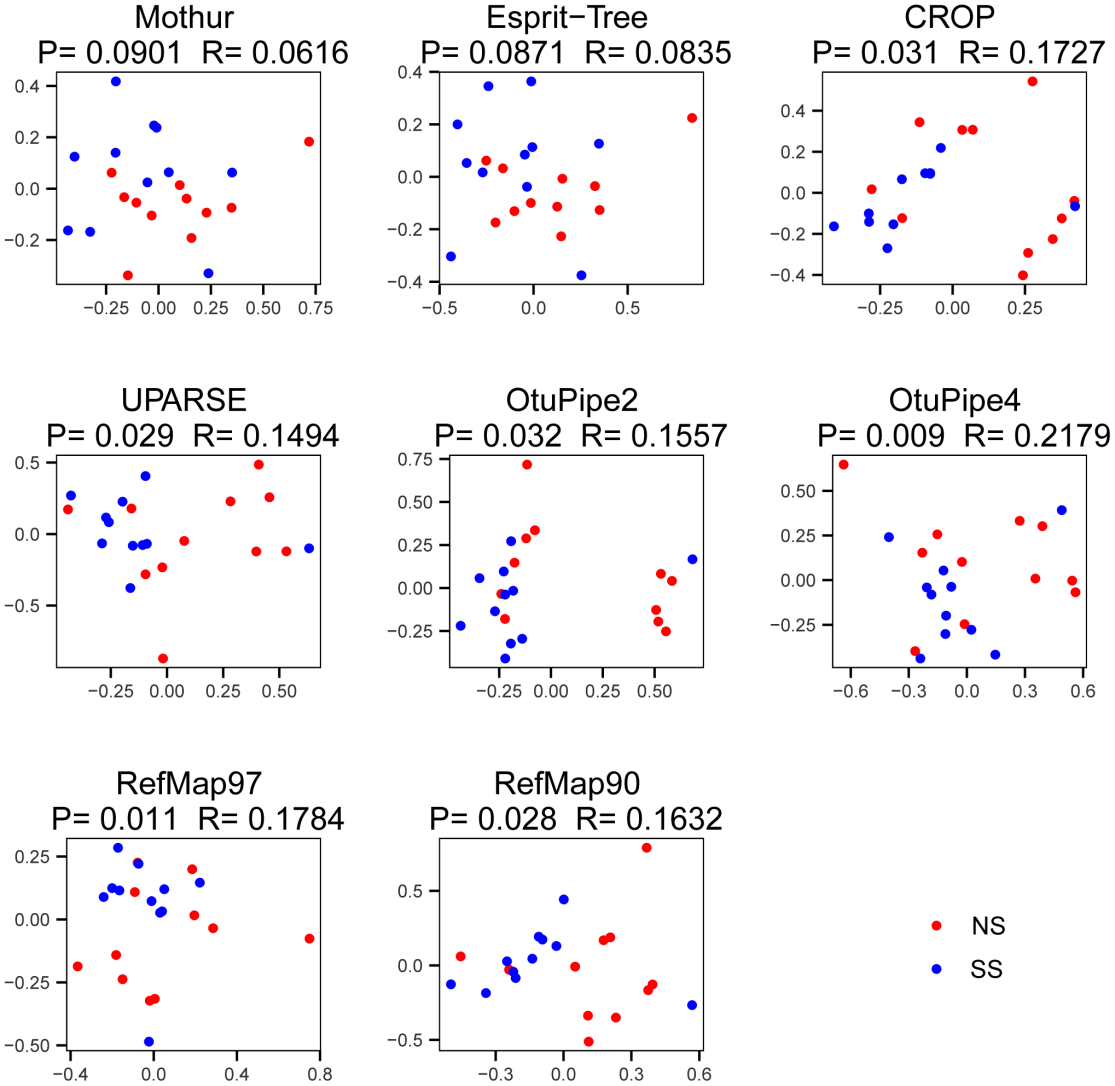
Non-metric multidimensional scaling plots of the distances for 22 fecal samples based on the different OTU matrices. Mothur, Esprit-Tree, CROP, UPARSE and Otupipe were used to bin 16S pyrotags into OTUs. Otupipe2 and Otupipe4 matrices were generated with MinSize parameter set to 2 or 4, respectively. In addition, 16S pyrotags were mapped into against a full-length 16S reference dataset to generate OTU matrices at 97% (RefMap97) or 90% (RefMap90) identity. The plots contain data from super-shedders (SS) and non-shedders (NS). ANOISM was used to test for similarities between SS and NS steers, generating correlation (*R*) and significance (*p*) values.

The SS and NS groups formed separate clusters on NMDS ordinate plots (Figure 3) for the majority of OTU matrices generated in this study. Analysis of similarity (ANOSIM) further confirmed these differences for six of the eight OTU matrices: CROP, UPARSE, Otupipe2, RefMap90 and RefMap 97; (*p*≤0.05) and Otupipe4 (*p* ≤0.01). These results provide strong support that the fecal bacterial communities of SS and NS groups had greater inter-group than intra-group differences. Furthermore, the Bray-Curtis distance was significantly larger within NS group than SS group: 1.2 and 0.9 respectively (one tail t-test *p*<0.01). Collectively, these results support the contention that SS and NS clearly harbored different fecal bacterial communities.

The Mantel test was used to examine the similarity of distance matrices generated by the different OTU methods; all matrices were highly correlated to the RefMap97 (*p*<0.001), but to different degrees (Table 1): Otupipe4 was the most similar, followed by RefMap90, UPARSE, Otupipe2, CROP, Mothur and Esprit-tree. Therefore, the Otupipe2 matrix was considered as the most relevant OTU matrix to differentiate taxa between super shedder and non-shedders owing to its high correlation with the RefMap97 matrix and its ability to cover >83% of processed sequences (Table 1).

### Identification of OTUs exhibiting differential abundance in super-shedders and non-shedders

**Figure 4:**
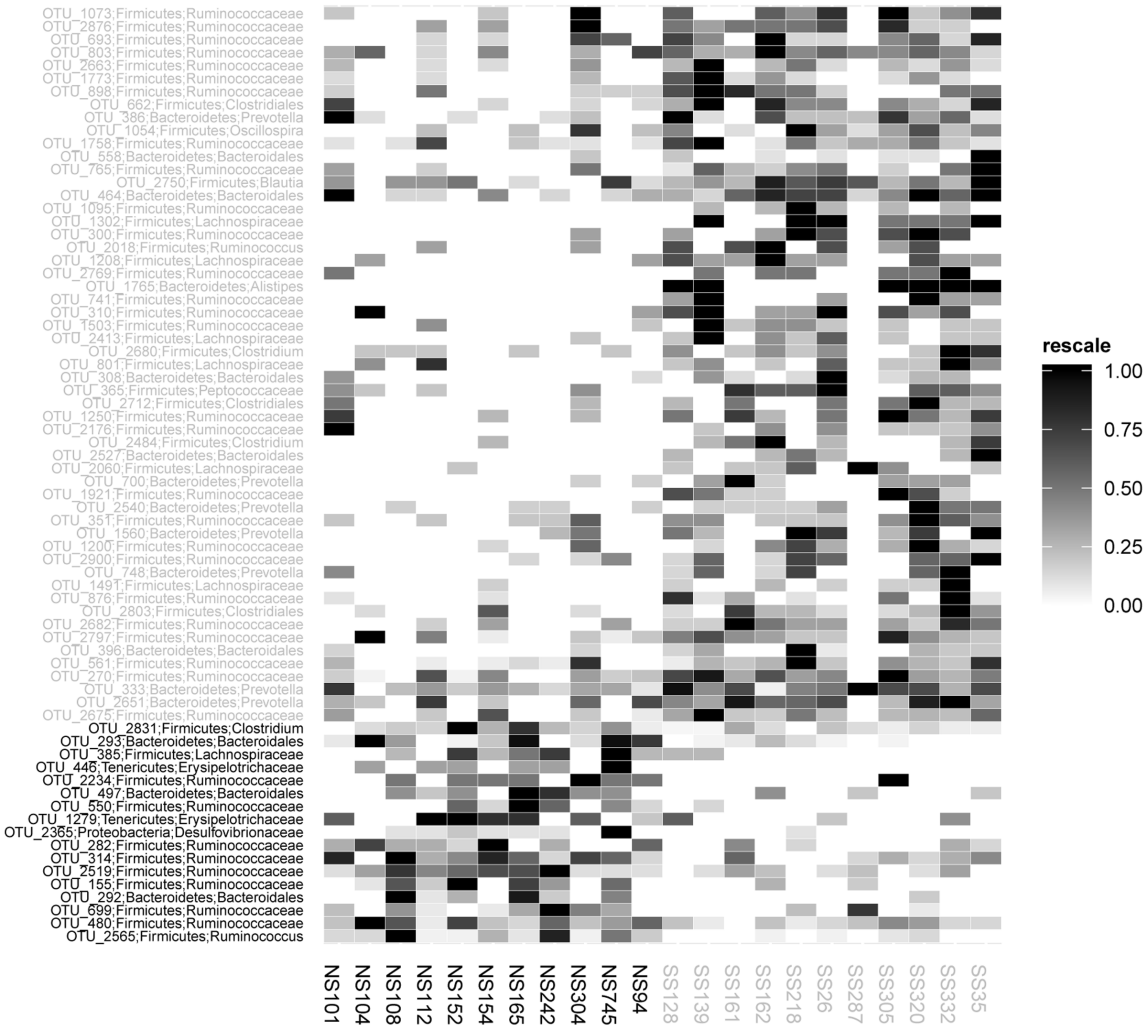
Heat map showing OTUs with a significant differential abundance (*p*<0.05) between super-shedder (SS; light grey axis labels) and non-shedder (NS; black axis labels) steers. Bacterial taxonomic affiliations are indicated on the left of the heat map at the phylum and genus level except in cases where genus is unknown in which case taxa are summarized at higher ranks. The relative abundance of each OTU is coded as indicated by the scale on the right, with highest number of pyroseqs for each OTUs set as 1.

Comparison of DNA extracted from fecal samples arising from SS and NS cattle revealed global significant differences in bacterial community structure. The differential abundance of OTUs was determined with the Otupipe2 OTU matrix and 72 OTUs showed differential abundance (*p*<0.05) between the SS and NS (Figure 4). Seventeen OTUs (23.6%) were enriched in NS, whereas 55 (76.4%) were more abundant in SS. These OTUs included *Firmicutes* (n = 53), *Bacteroidetes* (n = 16), *Tenericutes* (n = 2) and *Proteobacteria* (n = 1) (Table S2).

Of the differentially abundant *Firmicutes* OTUs, all were assigned to the *Clostridiales* order, with four known families represented: *Ruminococcaceae* (36 OTUs), *Lachnospiraceae* (10 OTUs), *Clostridiaceae* (3 OTUs) and *Peptococcaceae* (1 OTU). Three OTUs (OTU_662, OTU_2712, and OTU_2803) remained unclassified at the family level. Eleven OTUs were significantly more abundant in NS compared to 42 OTUs in SS. Among OTUs that were classified at the genus level, *Ruminocococcus* OTUs were associated with both NS and SS; OTU_2565 was more often found in NS with the opposite response found for OTU_2018. Of the three *Clostridium* OTUs, two (OTU_2484 and OTU_2680) were more frequently found in SS while the third (OTU_2831) was more abundant in NS. The single *Blautia* (OTU_2750) and *Oscillospira* (OTU_1054) OTUs were more often present in SS.

For the *Bacteroidetes* phylum, all were classified in the *Bacteroidales* order, with the remaining two OTUs not being classified beyond the phylum level. Thirteen OTUs were significantly more abundant in SS compared to 3 OTUs in NS. Eight OTUs classified at the genus level were preferentially abundant in SS: with seven OTUs assigned to *Prevotella* (OTU_333, OTU_386, OTU_700, OTU_748, OTU_1560, OTU_2540 and OTU_2651) and one to *Alistipes* (OTU_1765).

Differentially abundant OTUs identified as *Tenericutes and Proteobacteria, were* represented by two OTUs (OTU_446 and OTU_1279) and one OTU (OTU_2365), respectively. These OTUs were more abundant in NS steers.

## Discussion

In this study, 2.8% of the steers were super-shedding at the time of sampling. This is consistent with findings by Stephens et al., [32] whereby <10% of cattle in a herd were identified as super-shedders. However, in other studies, prevalence has been documented at over 20% [33]. The duration that an individual continues to express the super-shedding trait is unknown and intensive sampling of individual cattle has shown that the levels of *E. coli* O157:H7 in feces are highly variable [34]. At this point it is not known as to what extent this variability may arise from shifts in the resident microbial communities within the digestive tract.

Numerous studies have demonstrated a correlation between microbiota and host health including obesity [35], [36], brain development [37] and intestinal disease. In this study, we demonstrated significant differences in fecal bacterial communities between SS and NS cattle. Global differences in the microbiota in feces from SS and NS cattle were clearly visualized by their separation by NMDS based on five different OTU binning methods and eight OTU matrices (Figure 3). Although different OTU binning methods led to very different results, the general inference remained identical, increasing the robustness of our conclusions. We observed an increased richness (i.e., number of observed OTUs) of fecal bacterial communities in steers shedding high levels of *E. coli* O157:H7 and identified 72 OTUs showing significant differential abundance between SS and NS (Figure 4). Contingent upon their ability to be isolated, these bacterial species could potentially be good candidates for genomic analyses and co-culture studies with *E. coli* O157:H7 to explore possible mechanisms of bacterial interaction.

The statistical power needed to detect differential features for OTUs relies heavily on the number of sequences representing each OTU. Thus it is not surprising that most differentially abundant OTUs (i.e., 69 out of 72) are members of the two dominant phyla (*Firmicutes* and *Bacteroidetes*), which combined accounted for 89.5% of the dataset. However, the less abundant taxa may also play a role in the development of the SS state. Two OTUs from the phylum *Tenericutes*, which accounted for 0.2% of the total data, were also identified as differentially abundant between SS and NS steers. Although with the current sequencing depth, we can see a general trend of alpha and beta diversity between SS and NS, it is still difficult to recover all of the differentially abundant features. Despite the high sequencing depth (average of 10,000 sequences per sample) and Good's coverage (average of 86.9%), more than half of the OTUs were represented by less than 5 sequences.

**Figure 5:**
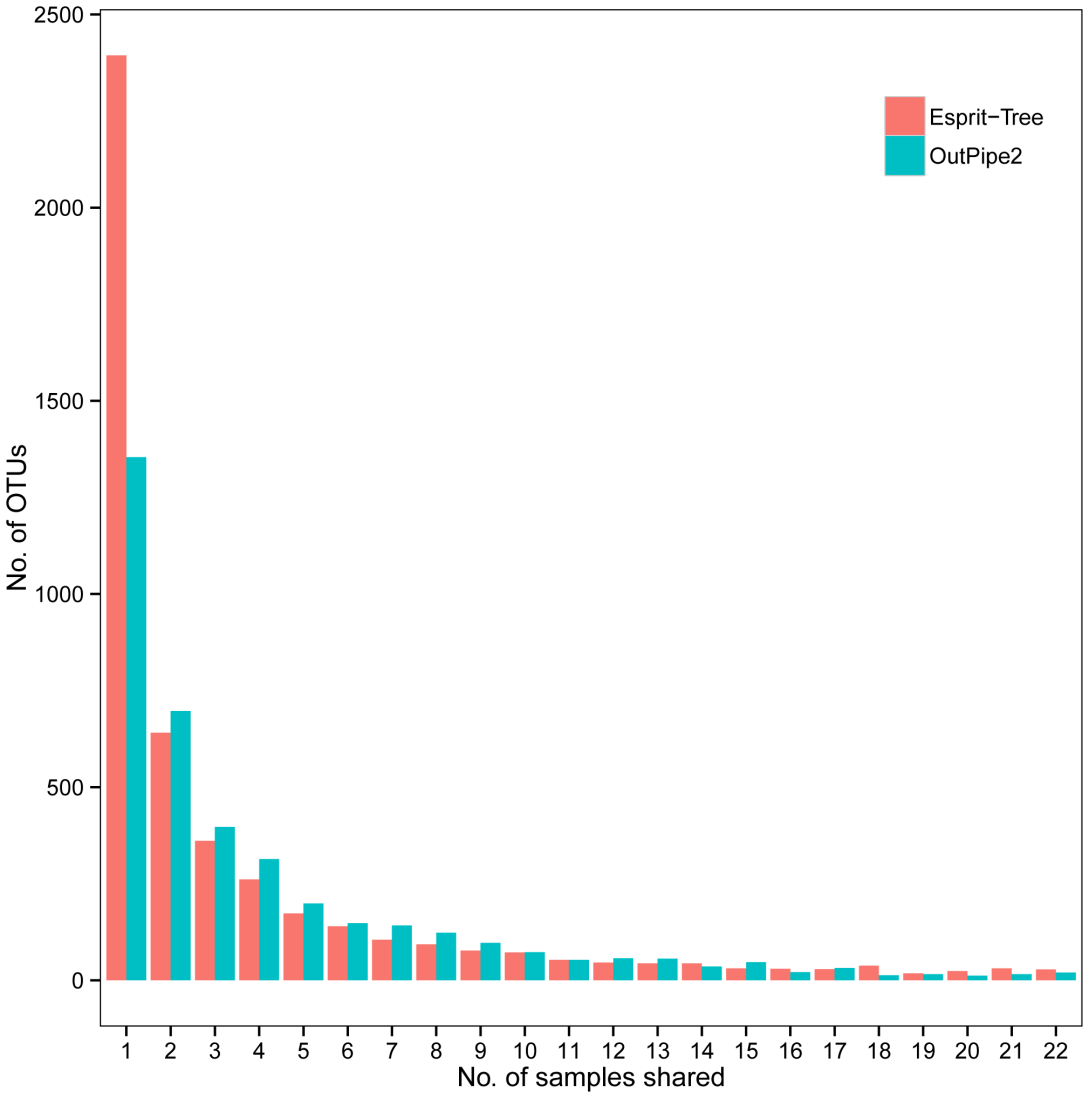
The number of OTUs shared by fecal samples.

Microbial populations in cattle feces are highly diverse from individual to individual. Our dataset was estimated to cover more than 85% of the bacterial diversity present in feces (Figure 2). The bacterial community in all steers was dominated by *Firmicutes*, accounting for 53.9%, followed by *Bacteroidetes* at 35.6% of total sequences, a pattern observed by others [11], [12], [38]. With this depth, however, only a small proportion of OTUs (<1%, or 10–50 OTUs) were shared by all 22 samples at 97% similarity level (Figure 5; Table S3). Shanks et al. [12] obtained similar results with fecal samples from 30 cattle using 16S rRNA gene pyrosequencing, in which only 9/9,201 OTUs (0.01%) were shared among all samples at a similarity level of 97%. In another study using full length Sanger sequencing of 16S rRNA gene, Durso et al., [11] identified 24 common OTUs out of 1, 906 OTUs (1.3%) at a 97% similarity level in six cattle fecal samples. Good's coverage and the rarefaction curve showed that the majority of OTUs in this study were captured in the sample analysis (Figure 2), indicating that the small number of shared OTUs is not due to a lack of sequencing depth. Therefore, there is an inherent high degree of variation in the bacterial community in cattle feces, but they clearly differ from those in the feces of humans, chicken, swine, geese or ducks (Figure S2).

In *E. coli* O157:H7, two systems including the locus of enterocyte effacement (LEE), responsible for establishing attachment and effacement lesions at the recto-anal junction, and the glutamate decarboxylase (gad) acid-resistance system, have been shown to play a critical role in intestinal colonization in cattle [39], [40]. Enterohaemorrhagic *E. coli* (EHEC) use several quorum sensing systems for intercellular signaling including the LuxR homolog SdiA that senses acyl-homoserine lactones (AHLs) which are present in the rumen but not in the lower GIT of cattle as the pH there does not favor AHL [41]–[44]. Although EHEC do not produce AHL, it is required for colonization of the GIT as it binds and stabilizes SdiA protein. Chemical signaling through SdiA-AHL promotes the survival of EHEC within the ruminant gut as it activates the expression of gad which aides in survival within the acidic rumen and conserves energy by repressing expression of LEE [45], [46]. Certain members of the *Bacteroidetes* have been reported to produce AHLs [47] and it would be interesting to investigate if the bacteria associated with OTUs depicting differential abundance are AHL producers.

Recent studies revealed the profound impact of diet on microbial community composition in cattle. For example, the abundance of the family *Ruminococcaceae* of the phylum *Firmicutes* and the genus *Prevotella* of the phylum *Bacteroidetes* were correlated with dietary change [12], [48], [49]. In our study, all steers were fed the same diet suggesting that community structure differences between SS and NS are linked to factors other than just diet composition.

A large number of studies [11], [12], [38], [49], including this one, have observed a high level of inter-animal variability in microbial intestinal communities. This phenomenon coincides with the possibility that conditions within intestinal microbial communities that are conducive to the shedding of high numbers of *E. coli* O157:H7 only occur in a limited number of cattle within a herd. The NMDS plots (Figure 3) show that bacterial communities from SS clustered together more so than those from NS, although some outliers were evident. The more similar microbial community structures within SS cattle suggest that this specific microbial composition may have allowed *E. coli* O157:H7 to proliferate and flourish. Perhaps the changes in microbial community composition in SS cattle result in differential degradation of organic matter, leading to a nutritional environment that is more favorable for the proliferation of *E. coli* O157:H7. Indeed, this enteric pathogen has evolved a number of unique nutritional pathways that may enable it to occupy a niche different from commensal generic *E. coli*. For example, *E. coli* O157:H7 strains are more likely to use dulcitol, sucrose and L-galactonic acid δ-lactone as carbon sources than other commensal *E. coli* [50]. In addition, *E. coli* O157:H7 can utilize free ethanolamine in the bovine small intestine as a nitrogen source, a gene cluster that is generally absent in the genomes of most of other bacterial species within gut microbiota [51]. The presence or absence of ethanolamine utilizing bacteria could also be a contributing factor towards the existence of distinct microbial communities between SS and NS cattle.

Another hypothesis to explain the differences between SS and NS cattle takes into account competitive inhibition mechanisms among *E. coli* strains [52], [53]. Some *E. coli* strains are able to inhibit the growth of others, resulting in shifts in *E. coli* populations that could influence the density of *E. coli* O157:H7 within the GIT. In this study, *E. coli* sequences represented only 0.015% of the dataset (17 pyrotags) and 16S pyrosequencing analysis was unable to discriminate between strains of *E. coli*. Further comparative studies focusing on *E. coli* populations could be useful to evaluate the importance of commensal stains in SS and NS cattle. Interestingly, the genome of *E. coli* O157:H7 is 25% larger and contains ∼1400 more genes than generic *E. coli* K-12 [54]. Among these genes, many are related to virulence but the function of >60% is unknown, but they may confer specific traits that increase the competitiveness of *E. coli* O157:H7 within the GIT of cattle. The recent sequencing of 26 *E. coli* O157:H7 strains may aide in the identification of gene clusters that contribute to its prevalence within the GIT of cattle [54].

In conclusion, significant differences in the composition of fecal microbiota of *E. coli* O157:H7 SS and NS cattle were identified, highlighting (i) a more diverse microflora in SS animals and (ii) specific bacterial OTUs that may be associated with the super-shedding state in cattle. These findings strongly support an important link between the intestinal microbial community and the density of this foodborne pathogen in cattle. However, whether *E. coli* O157:H7 overgrowth in super-shedders is promoted by intestinal dysbiosis or leads to bacterial population shifts remains unclear. Based on our results, future work could attempt to answer this question.

## Supporting Information

Figure S1 **Sequence data summary.** Left: Relationship between processed sequence length (cleaned) and the number of sequences, Middle: Comparison of aligned sequence length and sequence length frequency, Right: The number of pyrotags for each library in super-shedding and non-shedding animals.
(TIF)

Figure S2 **A Non-metric multidimensional scaling (NMDS) plot generated using fecal samples from different animal species.** Cattle_leth denotes 22 cattle fecal samples from this study. The other metagenomic data were downloaded from http://trace.ncbi.nlm.nih.gov/Traces/sra/?study=ERP000189.
(TIF)

Table S1 **Taxa at five taxonomic levels (phylum to genus) for all samples combined.** Total number of sequences for each taxa are shown in table.
(XLSX)

Table S2 **List of 72 differentially abundant OTUs**.
(XLSX)

Table S3 **The number of OTUs shared by fecal samples as determined by the various clustering methods.**
(XLSX)
