# Supplementary figures and images for: Escherichia coli O157:H7 Super-Shedder and Non-Shedder Feedlot Steers Harbour Distinct Fecal Bacterial Communities
Source: PLoS One. 2014 May 23;9(5):e98115. doi: 10.1371/journal.pone.0098115 (PMC4032279; doi:10.1371/journal.pone.0098115)

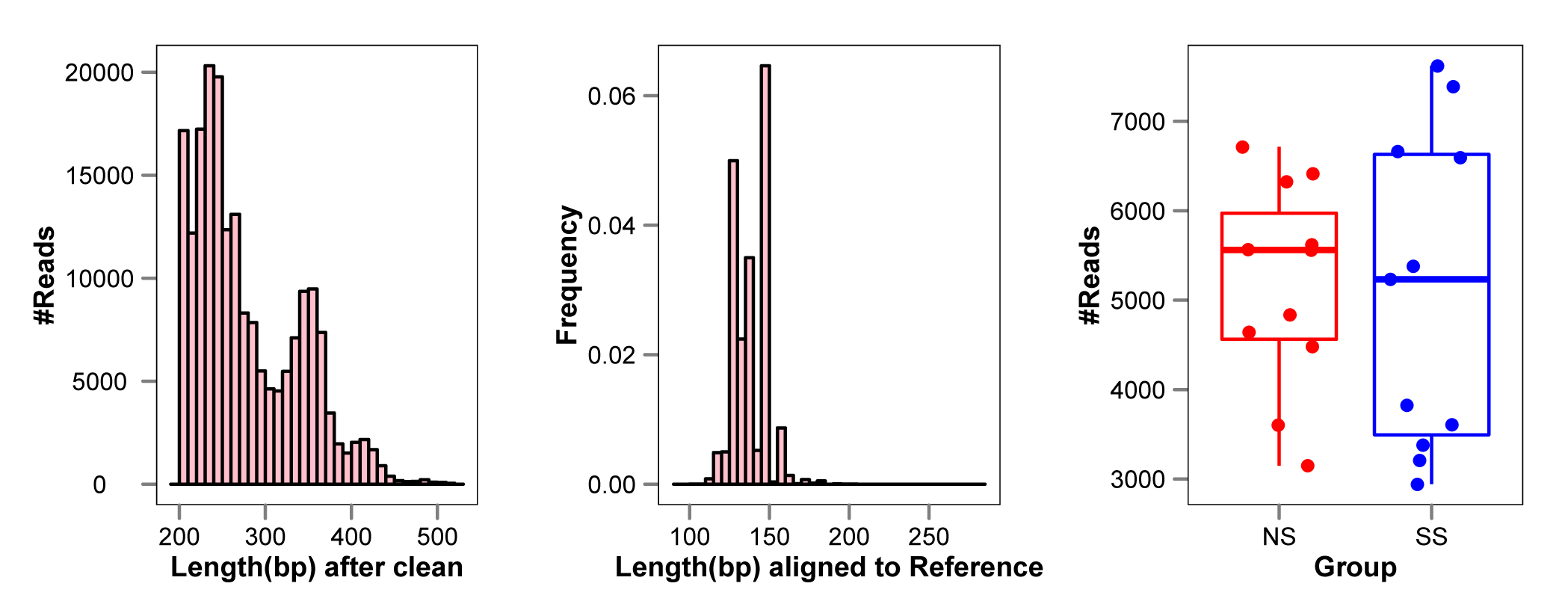

Supplement: Figure S1 — Sequence data summary. Left: Relationship between processed sequence length (cleaned) and the number of sequences, Middle: Comparison of aligned sequence length and sequence length frequency, Right: The number of pyrotags for each library in super-shedding and non-shedding animals. (TIF) [file pone.0098115.s001.tif]

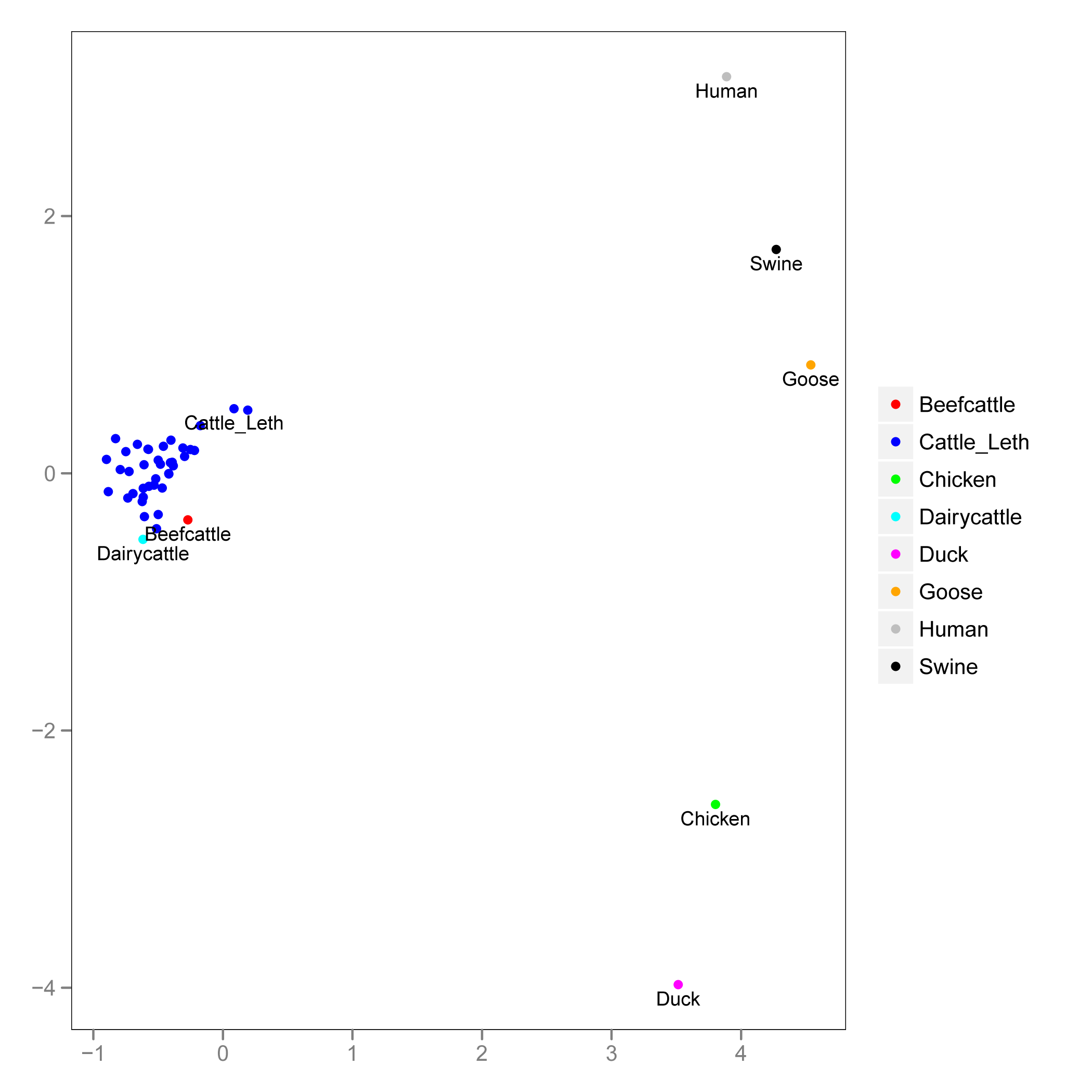

Supplement: Figure S2 — A Non-metric multidimensional scaling (NMDS) plot generated using fecal samples from different animal species. Cattle_leth denotes 22 cattle fecal samples from this study. The other metagenomic data were downloaded from http://trace.ncbi.nlm.nih.gov/Traces/sra/?study=ERP000189. (TIF) [file pone.0098115.s002.tif]
